# The Middlesex Hospital Appeal

**Published:** 1910-07-02

**Authors:** 


### Institutional Letter-Box.

### THE MIDDLESEX HOSPITAL APPEAL.

Sir,—In a letter which appeared in the Press on the 3rd inst. I appealed to the public for £20,000 in support of the Middlesex Hospital. For over a century and a half this great institution has done its splendid work of ministering to the suffering poor.

The Bishop of London has written me a letter, in which he says that it would be a great blow to London if anything happened to the old Middlesex. The keynote of its management is efficiency with economy. It stands in the very heart of the world's richest metropolis, but its career of activity and usefulness is seriously imperilled by diminishing income, and money must be speedily found to wipe off the existing debt. The sum of £20,000 is immediately required to save the hospital from a dangerous position.

I rejoice to say that the response to my first letter of appeal has been both prompt and generous. The total already amounts to several thousand pounds, and I feel greatly encouraged in the task I have undertaken. But the present need is very real and urgent, and I am compelled to redouble my personal efforts. The task is not an impossible one, and I have set my heart on accomplishing it. I know I am not appealing in vain, and with the generous aid of the Press and the public I confidently hope to succeed.

Let rich and poor alike give whatever they can spare; I will gratefully acknowledge the smallest contribution sent to me. To attain the object in view is not only my anxious care and ardent desire, but it is a matter of strenuous endeavour in which I call upon my fellow citizens to help me. "He gives twice who gives quickly."

I am, yours faithfully,
Francis of Teck, Chairman.

36 Welbeck Street, W., June 21.

